# School food programmes in the Pacific Islands: exploring opportunities and challenges for creating healthier school food environments

**DOI:** 10.1017/S1368980022001951

**Published:** 2022-11-21

**Authors:** Sarah Burkhart, Ann Hayman, Fiasili Lam, Breanna Jones, Bridget Horsey, Dana Craven, Steven Underhill

**Affiliations:** 1 Australian Centre for Pacific Islands Research, University of the Sunshine Coast, Locked Bag 4, Maroochydore, Queensland 4557, Australia; 2 School of Health and Behavioural Sciences, University of the Sunshine Coast, Maroochydore, Queensland, Australia; 3 Food and Agriculture Organization of the United Nations, Subregional Office for the Pacific Islands, Samoa

**Keywords:** Education, School food, Sustainability, Nutrition, Children, Oceania, Health

## Abstract

**Objective:**

The school setting can provide an environment that supports healthy behaviours, including the provision of food. School food activities, that is, school feeding, are commonplace globally, but not well understood in the Pacific Islands region. The aim of this research is to explore learnings associated within existing school food programmes (SFP), and adoption resistors in those Pacific Island Countries and Territories (PICT) without SFP, with the intent of improving current and future SFP interventions.

**Design:**

This observational cross-sectional study utilised four facilitated workshop sessions to explore SFP within an existing framework.

**Setting:**

Pacific Islands region.

**Participants:**

Fourteen participants representing the education and health sectors from eleven PICT, and two participants representing regional organisations.

**Results:**

Most countries reported some form of related policy, but key critical constraints to the use of SFP included local food environments, strategic alignment to organisational priorities, advocacy and organisational leadership, and community and cultural connections and collaboration. There are opportunities for integration of SFP into existing frameworks (i.e. Health Promoting Schools), increased collaboration, greater professional development and awareness activities, improved monitoring and evaluation, improved awareness of SFP and promotion of healthy eating for the wider school community.

**Conclusions:**

Given the current health, social and economic challenges faced by countries and territories in the Pacific Islands region, SFP should be considered as an opportunity for food provision and associated nutrition education for students and their wider community. Further research is needed to understand the critical constraints of SFP in this region and how to support stakeholders to advocate for, develop and sustain SFP that are contextually and culturally appropriate.

Nutrition transitions are underway in countries and territories throughout the Pacific Islands region as rapid urbanisation, globalisation and international trade bring about a shift from traditional subsistence-sourced food to an increased reliance on imported, and often highly processed foods^(1,2)^. Fresh food accessibility in the Pacific, particularly for fruits and vegetables, are often constrained by seasonality supply, low horticultural productivity, post-harvest loss, and adverse weather and climate impacts^(3–5)^. These changes have significantly impacted the health of these populations, with the triple burden of malnutrition (overweight and obesity, undernutrition and micronutrient deficiencies) observed in communities throughout this region^(6)^. Rates of overweight, obesity and diet-related non-communicable diseases (DR-NCD) in Pacific Island Countries and Territories (PICT) are some of the highest worldwide. In 2019, seven of the top ten countries worldwide for rates of adult overweight and obesity, and nine of the top ten children and adolescent (5–18 years of age) obesity rates were in this region^(7)^. At the same time, rates of stunting in children under 5 years of age are as high as 48·4 %^(8)^.

It is well recognised that nutrition status during childhood affects health in adulthood. Overweight and obesity, micronutrient deficiency, stunting and wasting during childhood increase the risk of other health complications and can compromise cognitive and social development in later life^(9–11)^. It is also recognised that the eating habits that children learn during an early age are likely to be carried into adulthood^(12,13)^. The health implications of poor nutrition status and DR-NCD place a considerable strain on the already limited health systems and economies of these PICT^(14)^. These countries and territories are reported to spend a greater proportion of gross domestic product (GDP) on health systems, with this likely to increase in the future^(15)^. Given the potential health, economic and social impact of a generation growing up malnourished, childhood and adolescence is an important period for intervention.

As children and adolescents spend a considerable amount of time in educational settings, schools are an opportunistic setting for intervention with this group. Schools and churches have an important role in Pacific society, influencing societal behaviours and attitudes^(16,17)^. Schools play an important role in providing education and are an avenue for the promotion of basic human rights, including the right to adequate food and the highest attainable standard of health, particularly for those in marginalised groups^(18)^. The school setting provides an opportunity to provide an environment that supports healthy behaviours^(19–21)^, including the provision of food and complementary nutrition education^(22)^. Additionally, schools provide a starting point for educating and supporting their wider community to adopt healthier eating behaviours.

Targeted nutritional interventions such as school feeding programmes are regularly implemented throughout the world, particularly in low- and middle-income countries to address undernutrition and learning outcomes in children^(23,24)^. School feeding programmes vary in approach taken based on need and desired outcomes; however, they usually include the provision of food to students as in-school meals and/or snacks or take-home rations^(25,26)^. School feeding programmes can help to alleviate short-term hunger, improve nutritional status, and enhance both academic performance and school attendance^(23,27)^ and can therefore play an important role in working towards several Sustainable Development Goals (SDG)^(28)^, including SDG’s 1 (No Poverty), 2 (Zero Hunger), 3 (Good Health and Wellbeing), 4 (Quality Education), 5 (Gender Equality), 8 (Decent Work and Economic Growth), 10 (Reduced Inequalities), 12 (Responsible Consumption and Production), 14 (Life Below Water) and 15 (Life On Land). Several strategies including policy implementation^(25)^ and alignment with educational activities that promote behaviour change in the short and long term^(18,22,29,30)^ are likely to enhance the success and sustainability of school feeding programmes. School feeding programmes can be a successful vehicle for providing access to food; however, these feeding programmes can require a significant investment^(31)^. When a formal school feeding programme is not in place, other strategies and activities (e.g. policy that supports healthier food choice, educational activities and school food events) on their own or as part of a framework can still help create a healthier school environment.

In the context of high rates of DR-NCD in the Pacific region and very high rates of school participation where most children attend school from the age of 5 years to at least mid-teenage years (16 years of age), school feeding programmes provide a unique opportunity to provide food and educate and motivate Pacific Island students and the wider Pacific Island community on the importance of healthy, sustainable diets. While school feeding programmes are established in many areas of the world^(23,27,32)^, their use is limited and not well understood in the Pacific Islands region, most likely because of limited policy, knowledge, partnering and implementation capacity^(33)^. While recent information on the current state and capacity for school food and nutrition activities in some PICT is available^(33,34)^, and school food environments in Fiji^(35)^ and Tonga^(36)^ and school food policy in Samoa^(37)^ have been investigated, an initial exploration of the broader concept of school food programmes (SFP) in this region is warranted. Understanding stakeholder perspectives and exploring opportunities and adoption resistors can provide useful evidence for targeted advocacy efforts. Therefore, the aim of this research is to explore learnings associated within existing SFP, and adoption resistors in those PICT without SFP, with the intent of improving current and future SFP interventions.

## Methods

This observational cross-sectional study reports on a workshop where school food stakeholders explored opportunities for and challenges to the use of SFP within an existing framework^(22)^ to complement current understanding of status and capacity for SFP in the Pacific Islands. This workshop was a component of a larger project, investigating the current state and capacity for SFP in fourteen Pacific Island countries^(33)^. Prior to the workshop, the research team had surveyed stakeholders (including Ministries of Education or (similar)/Ministries of Health (or similar), The Pacific Community (SPC) and individual researchers) about SFP, of which the findings were used to guide the content and goals of this workshop. In addition, a complementary project was undertaken in the year prior (2018), investigating the current state and capacity for school nutrition education programmes^(34)^ in the same fourteen Pacific Islands countries.

The authors recognise that there are several descriptions that can be used to define the provision of food in schools. For this study, a SFP was defined as an activity/intervention that aimed to provide access to or influence food in or near the school environment. For example, this could be through the provision of lunch, or another meal/snack while the student is attending school (typically considered a school feeding programme) or other *ad hoc* provision of food in schools, that is, using a canteen or ‘tuckshop’. It also includes other school food-related programmes, such as the use of policy to inform availability of foods at, or near the school premises and integration of food and nutrition education linked to food provision (i.e. gardening) and skill development where access to food occurs (i.e. cooking classes).

### Data collection

#### Stakeholders

Stakeholders from fourteen countries (Cook Islands, Federated States of Micronesia, Fiji, Kiribati, Niue, Nauru, Marshall Islands, Palau, Samoa, Solomon Islands, Tokelau, Tonga, Tuvalu and Vanuatu – all members of the FAO of the UN Subregional Office for the Pacific Islands) were invited to take part in the SFP workshop. Representation was also sought from The Pacific Community to gain a broader view of SFP in this region.

The Permanent Secretary/Director of each country Ministry of Education (or similar) and Ministry of Health (or similar) was invited via email to nominate a representative to attend a 2-d SFP workshop held in Nadi, Fiji, in August 2019. The Pacific Community was also invited to nominate and send a representative. The Ministries and SPC were part of the first stage of the larger project; however, the nominated representative was not necessarily the person who contributed to the preceding project survey. The invitational email outlined the purpose of the workshop and included a research participant information sheet outlining data collection and use. Workshop nominees were provided with background information on the project and an agenda prior to attendance. Workshop attendance was funded by FAO.

#### Workshop sessions

Four sessions were used to explore SFP; three sessions dedicated to discussion of the FAO School Food and Nutrition framework^(22)^ key areas of work, and one session focused on considerations of sustainability, cultural factors and a discussion on moving forward with SFP in the Pacific Islands. The questions used in each of these sessions were developed and reviewed by the research team.

#### School food programmes in the context of the FAO school food and nutrition framework

Three sessions were used to gather data on SFP and discuss country and regional SFP in the context of the FAO School Food and Nutrition framework^(22)^. These sessions were based on the four key areas of work of this framework: healthy food environment and school food; food and nutrition education; enabling policy, legal and institutional environment; and inclusive procurement and value chains. Stakeholders were provided with a definition of the relevant component of the FAO School Food and Nutrition framework^(22)^ at the start of each session. The fourth session focused on sustainability, cultural factors and forward-thinking. Each of these sessions is outlined.

#### Session one: healthy food environment and school food; enabling policy, legal and institutional environment

As a primer to providing individual responses, stakeholders were asked to consider and provide verbal responses to the questions in small groups; Does the food environment in your country support SFP? Why/why not? What types of foods can be sourced locally? How do these compare in cost to imported foods? What are the most significant challenges of the food environment in relation to SFP? What changes are needed in the food environment if SFP is to be successful? Stakeholders were then asked to individually consider and record responses to the following questions on a paper-based survey based on their country situation: What are challenges to the success of SFP that relate to the food environment and what are potential solutions? What activities are you currently doing that relate to this area? What activities would you like to do?

In small groups, stakeholders were asked to consider and respond to the questions: Who has a national policy to support SFP? How has this worked? What have been the challenges? Who has a school-level policy to support SFP? How has this worked, what have been the challenges? Who does not have any current policy to support SFP? Stakeholders were then asked to individually consider and respond to the following instructions on a paper-based survey based on their country situation: Please record any SFP-related policy you currently have in your country, and who are your key stakeholders for advocacy?

#### Session two: food and nutrition education

In small groups, stakeholders were asked to discuss and then make notes on a provided paper-based survey tool for responses to the questions: how is food and nutrition education integrated into your curriculum? What subjects include nutrition? Are teachers provided with training for nutrition-related content? Do you integrate gardening into the curriculum? If so, how?

#### Session three: inclusive procurement and value chains

An overview of home-grown school feeding^(38)^ was provided in addition to the School Food and Nutrition framework^(22)^ definition to provide context in this session. In small groups, stakeholders were asked to consider and respond to the following questions in a facilitated discussion: Are there opportunities to use models like home-grown school feeding in your country? Can you suggest other models that may work? Do you know of any examples of these? Who are the key stakeholders that need to be included?

#### Session four: sustainability and cultural considerations of school food program, moving forward

During this session, the following questions were posed to the group: What would help to ensure the sustainability of SFP activities in your country? And what cultural considerations need to be considered for SFP in your country? Stakeholders were asked to discuss these questions in small groups and add their responses to whiteboards provided at the front of the room. Additionally, during a facilitated discussion, stakeholders were asked to provide a verbal response to the question: Where do we go to from here when considering SFP in the Pacific Islands?

Members of the research team (*n* 2 two researchers) recorded notes from each session and collated other forms of evidence from stakeholders (i.e. paper-based surveys, comments added to a whiteboard). Notes were summarised and presented to stakeholders on both days of the workshop for verification. It was not practical to record the workshop due to venue limitations, and the research team believed that authentic conversation was more likely without participants feeling as though they needed to discuss and respond to questions in a particular way. A workshop summary document was developed and sent to all stakeholders 10 d after the workshop to provide further opportunity to verify findings. Any amendments provided by stakeholders were updated in the data prior to analysis.

### Data analysis

Participant responses were analysed using an inductive content analysis approach with three steps: preparation, organisation and reporting used^(39)^. During the preparation phase, three researchers immersed themselves in the data by reading through these multiple times^(39)^. During the organising phase, two researchers independently used open coding to apply initial descriptive code labels to the responses^(39)^ to highlight key thoughts or concepts^(40)^. Initial code labels were used to generate categories^(41)^ which were then abstracted to create key categories, providing a description of the topic^(39,41,42)^. Two researchers completed this process independently to enhance category refinement and trustworthiness. The researchers then discussed any dissonance in coding together to finalise the analysis. Data are presented collectively to preserve individual responses within the regions of Melanesia (Fiji, Solomon Islands and Vanuatu), Micronesia (Kiribati, Nauru, The Federated States of Micronesia, Republic of the Marshall Islands and Palau) and Polynesia (Niue, Samoa and Tonga).

## Results

### Stakeholders

Fourteen stakeholders from eleven PICT including Federated States of Micronesia (*n* 1), Fiji (*n* 1), Kiribati (*n* 2), Nauru (*n* 1), Niue (*n* 1), Palau (*n* 1), Marshall Islands (*n* 1), Samoa (*n* 3), Solomon Islands (*n* 1), Tonga (*n* 1) and Vanuatu (*n* 1) attended and participated in the workshop. Stakeholders came from education (*n* 9) and health sectors (*n* 5). Additionally, one representative from SPC and one from FAO Subregional Office for the Pacific Islands also participated. Most of the participants were female (*n* 10 females, *n* 5 males). Level of education was not gathered from participants; however, it is assumed that as all were working for Government Ministries or The Pacific Community that they were experts in the workshop topic.

### Session one: healthy food environment and school food

#### The local environment and challenges to the use of school food program

During group discussions, stakeholders generally agreed that their local environments can support SFP. However, despite some groups noting an abundance of fruits, vegetables and fresh fish, some stakeholders reported food availability and cost as challenges. Food availability was impacted by seasonality of supply. Cost and convenience were important as the increased availability of processed foods affected food prices (i.e. local foods could in fact be more expensive than imported items). For example, fish was highlighted due to locally caught fish being perceived as more expensive to purchase than imported fish (tinned/canned/frozen). Stakeholders reported that imported foods are ‘much’ cheaper and last longer but are unhealthy compared with local fresh foods. One group discussed the importance of encouraging people to think of imported foods as being more expensive due to long-term medical costs associated with outcomes of consuming these. Climate change and sustainability, lack of access to kitchen facilities and food safety practices were also highlighted as key challenges to the use of SFP.

Stakeholders described the difficulty that occurs when schools attempt to provide SFP when children prefer unhealthy foods and shop owners near schools actively promote these foods. An example of this was how water can be provided to students at lunch, but schools are not always aware of other drinks students bring onto and consume on school grounds. Stakeholders also highlighted that the school environment is not directly aligned to the students’ home environment as some parents are unable to afford healthy food and schools are not able to control what parents feed their children. Stakeholders believed that to be successful, addressing the challenges raised was important, but that *‘we need to change people’s mindsets to make SFP successful’* (Group 2).

#### Challenges to the success of school food program and possible solutions

Individual responses to the challenges faced included the key categories of financial resources; political commitment and high-level collaboration; policy and documentation; monitoring and evaluation; sustainability; advocacy, promotion and awareness; and food procurement and preparation and structural factors (Table 1).

**Table 1:** Challenges to the success of school food programmes presented by key category and relevant reported policy in Micronesia, Melanesia and Polynesia

Challenges to the success of SFP key category – example quote/s	Micronesia (Kiribati, Nauru, FSM, RMI and Palau)	Melanesia (Fiji, Solomon Islands and Vanuatu)	Polynesia* (Niue, Samoa and Tonga)
Financial resources - *‘funding is an issue’*	X	–	X
Political commitment and high-level collaboration - *‘lack/no political commitment – lot of talk, no action’; ‘need Ministry of Education to assign a committee for SFP’*	X	X	X
Policy and documentation - *‘Drafting the policies and process’; lack of documents’*	X	–	X
Monitoring and evaluation (including certification) - *‘Monitoring of the SFP related policies and its implementation’*	X	–	X
Sustainability - *‘Sustainability of program goal and plan’* - *‘sustainability/climate change’*	X	X	–
Advocacy, promotion and awareness - *‘Advocacy and communication at all levels’* - *‘need to research about SFP’*	X	X	–
Food procurement and preparation - *‘Increasing imported foods in country’* - *‘procurement laws tend to slow and limits ability to procure food items in a reasonable manner’*	X	–	X
Structural factors - *‘Lack of structure especially at the outer islands’*	X	–	–
Policy reported by stakeholders			
Country-specific Food Security, and/or Food Safety and Nutrition (National) policy and/or Food Act	RMI, Kiribati	Fiji, Solomon Islands, Vanuatu	Samoa, Tonga
General nutrition guidelines/city ordinances	RMI	Fiji	–
School food-related guideline, policy and/or programme	Kiribati, FSM, Nauru, RMI, Palau	Fiji, Vanuatu	Samoa, Tonga

* Responses from Samoa and Tonga only.

Stakeholders provided a list of solutions, and while these were not considered in direct response to the identified challenges, responses aligned to the key challenges identified, with the additional key category of collaboration emerging. For example solutions included; ‘*seeking specific assistance to fund the school feeding program and related activities’* (key category: financial resources), *‘lobby for political commitment’* and *‘assign a team/officers within the Ministry to focus on school food programs’* (key category: political commitment and high-level collaboration); ‘*review policies in place’* (key category: policy and documentation); *‘schools to activate or use their school committee to assist with the enforcement of selling healthy food’* (key category: monitoring and enforcement); *‘a forum platform to talk about food and nutrition security’* and *‘public awareness programs for parents and the community’* (key category: advocacy, promotion and awareness); *‘work with local vendors to ensure availability of supplies’* and *‘(find) easier ways to procure healthy, local food items’* (key category: food procurement and preparation); and ‘*a network partnership’* and *‘network and engage actively with partners and agencies’* (key category: collaboration).

#### Presence of school food program-related policy

Seven countries were reported as having country-specific Food Security, and/or Food Safety and Nutrition (National) policies, while two countries reported general nutrition guidelines/city ordinances, and nine countries a school food-related guideline, policy and/or programme (Table 1).

#### Current and desired activities

Similar key categories emerged when stakeholders provided responses to questions about current and aspirational (would like to do) activities. Stakeholders reported that SFP in their countries currently included: professional development and awareness activities; curriculum; food provision; policy and standards; food provision; monitoring and evaluation; and gardening. Aspirational activities were similar but also included: advocacy for and integration of SFP into the WHO Health Promoting Schools framework; collaboration and sharing of practice; and raising awareness of SFP and promotion of healthy eating (Table 2).

**Table 2:** Key activities and examples of current and aspirational school food programme (SFP) activities in Micronesia, Melanesia and Polynesia

Key categories of activities	Current and aspirational activities by region					
	Micronesia		Melanesia		Polynesia	
	Currently do (example quote)	Would like to do (example quote)	Currently do (example quote)	Would like to do (example quote)	Currently do (example quote)	Would like to do (example quote)
Professional development and awareness activities for the school community	*‘Providing teacher trainings on different school policies’*	*‘Hands on training for the cooks’*	*‘Nutrition talk to teachers and students’*	*‘Train teachers in nutrition topics’*	*‘Training and awareness sessions with school community’*	*‘Empower teachers and school officers to have clear understanding on SFP’*
Monitoring and evaluation (including compliance)	*‘School staff and members of the school committee occasionally check food sold out in the school compound’*	*‘A series of random un-announced visits to check and ensure SFP relevant policies and legislation are adhered to and act accordingly to address the issues/problems’*	*‘School visits to all schools annually- canteen inspection/garden inspection’*	–	*‘Ongoing monitoring of the schools on the standards’*	*‘Conduct ongoing monitoring’*
Policy and standards	*‘Collaborate with other ministries and organisations to develop programs and policies towards issues such as SFP’*	*‘Policies strengthened and implemented’*	*‘Provide food with the guidance of sweet drinks policy’*	–	*‘Food Act school/food policies and manuals’*	–
Food provision	*‘Have school feeding program receiving yearly budget from the national government’*	–	*‘Urban primary schools and all secondary schools (boarding and non-boarding) have own chefs and kitchen’*	*‘Further work on boarding school meals’*	–	–
Gardening	*‘Implementation of the learning garden program in schools’*	–	*‘School gardens (boarding schools)’*	*‘Involvement of basic agriculture (gardening) in primary schools’*	–	*‘Vegetable and fruit garden’*
Curriculum	*‘Incorporate health in the school curriculum’*	–	*‘(have) Input into curriculum’*	–	–	–
Raise awareness of SFP and promote healthy eating	–	*‘Raise awareness to the public on the importance of healthy eating’*	*–*	*‘Lobby to have food and nutrition taught for all students at secondary schools’*	*–*	*‘Create posters that promote healthy eating in schools and display around the canteen’*
Advocacy for and integration of SFP into Health Promoting Schools	–	*‘Incorporate SFP into* Health Promoting Schools*’*	*–*	*‘Identify school food program component that will fit in healthy promoting schools’*	*–*	*–*
Collaboration and sharing of practice	–	*‘Call for a meeting between education and health to collaborate on a comprehensive policy of food programs in schools’*	*–*	*–*	*–*	*–*

#### Challenges to the use of school food program-related policy

Key group discussion points were related to actual policy, the role of modelling behaviours, food availability and programme design. Stakeholders believed that policy needed to be broad and not primarily focused on children. Stakeholders reported that some policies in place do not directly refer to the school setting. There was an acknowledgement that cost and funding can limit policy development, and that translation of policy into action needs work and resources. Stakeholders reported that food vendors can be focused on making a profit at the expense of health and, therefore, do not follow the related policy(ies). Stakeholders also discussed the need for role modelling behaviours and consideration of food availability in policy development and use. Programme design challenges included implementation, monitoring and evaluation, which were all viewed as important aspects of policy and the enabling environment.

#### Key stakeholders for advocacy

Stakeholders identified nine groups of stakeholders for SFP advocacy including school advocates (school board/committee, Parent and Teacher Association, student council), teachers, parents/schools, students, government ministries & personnel, NGO’s, business owners, the community, and food stakeholders.

### Session two: food and nutrition education

#### Integration of food and nutrition education in the school curriculum

Food and nutrition education discussions centred on the inclusion of this topic across subject areas of the curriculum. Six stakeholders reported that their country included food and nutrition education at primary, secondary or both levels, while stakeholders from two countries noted that this was a work in progress and that consultation between relevant Ministries was underway, or more collaboration between departments was required. Nutrition as a subject was included in Health/Physical education at early childhood level in one country. One country stakeholder reported that specific aims and objectives were already in place in the curriculum, while another reported that the Ministry of Education seeks information from the Ministry of Health to integrate food and nutrition into the curriculum, and one used a curriculum planning team, coordinators and teacher consultants.

Nine countries reported that food and nutrition education was included in health and/or physical education, seven in home economics, four in science, three in agriculture science and one each in food and nutrition, and social sciences. There are queries about how much exposure students get to food and nutrition education, particularly if they can choose subjects during the higher years of education. Stakeholders also discussed that having an interest in the topic of food and nutrition is important for students and teachers.

#### Training for teachers

Two countries reported that teachers were not provided with training for nutrition-related content. Seven countries reported that training was provided; however, for three of these this was noted as being very basic training, while four reported that this training was provided by the school’s own professional development programme, the Ministry of Health, Ministry of Education, or other organisations including NGO’s and consultants. When provided, teachers reportedly take part in activities to upskill during the holiday period.

#### Use of gardening in the school curriculum

Methods to integrate gardening into the curriculum included specifically through the subjects of agriculture (*n* 5 countries) sometimes with the assistance of the agriculture sector, science (*n* 2 countries), environmental science (*n* 1 country), food and nutrition/health (*n* 1 country), and as extracurricular activities (*n* 4 countries). One country reported this was done using consultation between the health and education sectors and the Health Promoting Schools Diet and Physical Activity training component. Stakeholders discussed the use of gardening (including fruit trees) in different subjects, and how this was often used in boarding schools. Stakeholders also identified that parents are an integral component of food and nutrition education.

#### Session three: inclusive procurement and value chains

There was a consensus that models like home-grown school feeding^(38)^ could be used in each country; however, there were country- and region-specific considerations discussed. Some stakeholders reported that parents play an important role in providing food for schools, sometimes in exchange for school fees. Parents support the school as an integral component of the local community that provides a service to their children. There was recognition that informal agreements may work, and there are benefits to these as they are done under the premise of good will, but there is a risk to formalising agreements, including reducing the goodwill that is already in place, and possible a decline in quality of food. No stakeholders were able to identify any specific examples of these models; however, they identified the key stakeholders for these models in their countries to be from the Ministry of Agriculture, parents, Ministry of Health, Ministry of Education, local vendors/small holder farmers, school Principal/board/Parent and Teacher Association, students, and school staff/gardeners.

### Session four: sustainability and cultural considerations, and where to next with school food program in the Pacific Islands

#### Sustainability

Strategies to ensure sustainability of SFP included alignment of SFP to higher-level priorities, plans, agendas and the SDG; ‘*We may have to link the SFP to our Government priorities/World Education Agenda/SDGs’ (Group 2).* Incorporating SFP into and strengthening the Health Promoting Schools programme was proposed. Stakeholders also noted the importance of extensive consultation with local communities and stakeholders to inform SFP programme development; ‘*Stakeholder involvement including community outreach programs to inform and get feedback for better implementation, monitoring, evaluation, way forward’ (Group 3).* Regular monitoring and evaluation on monitoring documents, as well as an increased awareness of relevant documentation, was proposed to increase ownership of these and support sustainability.

#### Cultural considerations

Cultural considerations for SFP were focused on the engagement and involvement of community and church leaders and groups to ensure beliefs and practices were considered and healthy eating/lifestyles were enforced in cultural activities and gatherings; ‘*Utilise community and cultural affairs to help and support SFP regulations (example, regulating food consumption, connecting cultural norms and eliminating NCD related behaviours/risks)’ (Group 1).* Stakeholders suggested that local and community land could be utilised for school gardens.

#### Where to next?

Stakeholders believed there is great potential for SFP in the Pacific Islands region, for example, *‘SFP can be the way forward’* and *‘[Country X] has focused more on WASH, but there is a need to bring SFP into the agenda’* (WASH refers to Water, Sanitation and Hygiene). However, consideration of other existing programmes and complementary activities is crucial in moving forward; *‘any discussions moving forward need to consider the Health Promoting Schools framework’, ‘if schools and teachers were expected to do this program, it cannot be an extra load, but something that is seen as complementary’, ‘let’s reinforce the programs that already exist’.* There was also a focus on being able to access relevant documentation; ‘*all the documents and policies need to be made available online or shared with everyone’.* Stakeholders expressed their gratitude for being able to share and collaborate with other like-minded individuals; ‘*this was a very important workshop and it was great to see what other countries are doing that is SFP related*’ and ‘*after returning home learnings will be shared*’. A desire to be responsible for and to contribute to advocacy and empowerment was also highlighted for example; *‘if not us, then who?’[*will advocate for and make SFP happen*]* and *‘if not now, then when?’,* as well as *‘when going back home there is a need to strengthen and empower everyone’* and we need to *‘help make education embrace SFP’*. Stakeholders also saw an opportunity for local foods to be incorporated into SFP; *‘there could be a good link between healthy school food and local production’* and *‘the synergies with a focus on local foods’.*

## Discussion

This study aimed to explore learnings associated within existing SFP, and adoption resistors in PICT without SFP with the intent of improving current and future SFP interventions. Given the current health, environmental, social and economic challenges faced by countries and territories in the Pacific Islands region, SFP should be considered as an opportunity for food provision and associated nutrition education for students and their wider community. There are opportunities for the integration of SFP into existing frameworks, increased collaboration, greater professional development and awareness activities (particularly for teachers), improved monitoring and evaluation, and improved awareness of SFP and promotion of healthy eating for the wider school community. However, key critical constraints to the use of SFP identified in this study were related to local food environments, community and cultural connections, strategic alignment of SFP to organisational priorities and policy, advocacy and organisational leadership, and collaboration.

There was consensus that local food environments are somewhat supportive of SFP; however, stakeholders also identified several factors that may constrain the use and effectiveness of SFP. While several food items can be sourced locally, that is, fish, fruits and vegetables, there was concern about increased access and availability of highly processed, imported foods; however, we did not explore if this based on physical and/or economic accessibility. Evidence shows that physical availability of these highly processed foods has increased throughout some PICT in recent years^(1,2,6,43,44)^. Stakeholders discussed these foods in reference to convenience; however, we did not explore what this convenience looks like, that is, convenient access to or convenient for consumption. While stakeholders discussed highly processed foods in the context of procuring food for school meals and as what students are exposed to in, there is very little published literature describing Pacific Island school settings. In 2020, a mapping activity of the 400 m radius about 187 Fijian schools found that 80 % of the outlets sold sugar-sweetened beverages, 63 % sold confectionery and 57 % sold fried beans^(35)^. Previously Tongan school students have been reported to obtain ‘unhealthy’ foods from in and around school environments^(45)^, and more recently the prevalence of these foods outside Samoan schools has been reported as a concern^(37)^, but work in this area is limited. Further work to explore the environments within and surrounding schools, as well as children’s food behaviours (i.e. if they purchase foods out of school and determinants of food choice), would be of benefit in this region. Additionally, given that highly processed foods may be perceived as a less expensive option, further investigation of food procurement processes in schools and the presence of supportive nutrition standards is warranted.

Stakeholders expressed concern that while the school environment can be designed to be more supportive of healthy food choices, this was often at odds with the community and/or home environment. It can be difficult to encourage children and adolescents to adopt healthy eating behaviours if these are not modelled at home, or in their local community^(46)^. While a whole of school approach has been discussed in Australian literature^(47)^, the emphasis placed on cultural considerations and community by stakeholders in this study and other similar work across FAO Subregional Office for the Pacific Islands member countries and in Samoa^(34,37)^ suggests that a ‘whole of community’ approach may be more appropriate for SFP in the Pacific Islands setting. The importance of culture in Pacific Island communities necessitates that local involvement and ownership of SFP is priority. While stakeholders noted that some cultural norms, that is, feasting, do not necessarily support promotion of healthy eating behaviours, which is also acknowledged in Fotu *et al*.’s work in Tonga^(45)^, engaging community and church leaders in SFP discussion could facilitate a connection of cultural norms, behaviours and community wellbeing. Stakeholders also emphasised the need for professional development activities for both the school and wider community. Providing complementary nutrition education for parents and the wider school community may enhance the success of school-based activities. It would be of interest to explore communities to assess if school guidelines can be extended further into the community or community uptake of these guidelines. Community connection may also provide opportunities to connect local food production with the provision of food in schools and related activities (i.e. integration of food production and processing activities into the curriculum). While there is literature outlining the mutual benefits of community-based food production models, that is, home-grown school feeding^(30,38)^, no such information is available for the Pacific Islands. Further exploration of community involvement in SFP in the Pacific Islands region, particularly for boarding schools where significant parent/caregiver input has been identified, may help to identify models, that is, approaches to school/community gardens that may work in other PICT regions. It would also be of interest to investigate the feasibility of local community integration in and contribution to food and nutrition curriculum in this setting and if this is associated with food sovereignty.

Any future discussion and action towards SFP in this region needs to recognise and consider how SFP can complement existing activities, priorities and resource use. A similar project across the same fourteen countries reported that capacity for school food and school nutrition education programmes is constrained by policy, knowledge, partnering and implementation capacity^(34)^, which is likely due in some part to geographical location and access to human, physical and financial resources. Stakeholders in this study reinforced that links to existing programmes is crucial, citing that with limited resources it is important that *we don’t reinvent the wheel.* Strategic alignment of SFP to organisational priorities and policy was seen in the stakeholder’s desire to align to Health Promoting Schools. Many PICT were already using or were aware of the Health Promoting Schools framework and saw this as a strategic opportunity for integrating SFP^(33)^. The Health Promoting Schools framework includes a diet and physical activity component, which has been successfully used in other countries, including some PICT^(34,48)^. Health Promoting Schools also emphasises the interaction of the school, students, teachers and non-teaching staff, and community links^(49)^, which aligns to the need for significant community involvement in the Pacific Islands region. Further investigation of the potential of Health Promoting Schools and Pacific Island-specific resources and curriculum would be of benefit to stakeholders considering Health Promoting Schools as an avenue for the integration and promotion of SFP.

A high level of motivation for the use of school food and nutrition education activities is observed in this region^(33,34)^. Many stakeholders report using multiple and innovative food provision and nutrition education activities for schoolchildren and the wider community to promote healthy and sustainable diets, but these can be constrained by a lack of resources^(33,34)^. Several solutions to the identified challenges were proposed by stakeholders. However, stakeholders see challenges related to raising the profile of SFP on political and high-level agenda and gaining sustainable commitment for funding and policy, and effective monitoring and evaluation. This apparent disconnect between the high-profile issue of DR-NCD and the potential benefits of SFP may be due to a lack of high-level political awareness of the benefits of SFP or a lack of resourcing to support advocacy. In Samoa, Reeve et al identified school food was not prioritised by non-health stakeholders, impacting the effectiveness of school food policy^(37)^. With limited physical, financial and human resources to support healthy school food environments, there is a need for continued, collective advocacy to promote and raise awareness of the benefits of school-based food and nutrition activities, including SFP^(50)^. While there are several nutritionists working in PICT, the collaboration displayed between health, education and agriculture sectors demonstrates a diverse group of stakeholders who are already working collectively to raise the profile of food and nutrition in the school setting. It is recognised that investment in school feeding programmes can provide a significant benefit for every dollar invested^(31)^. This warrants further investigation, with activities such as a cost–benefit analysis a viable opportunity to demonstrate financial benefits of SFP in this region.

Collaboration was viewed as an essential component of SFP in the future, with stakeholders expressing a desire to participate in collaborative activities and to learn from sharing experiences. However, this appears to be limited due to geographical isolation, funding and human resources, and limited forms of communication. Specific examples of solutions were provided by stakeholders from Micronesia only, possibly reflecting the geographical isolation of these countries. We found that monitoring and evaluation presents a significant challenge in this region, and that knowledge capacity is somewhat limited. Reeve *et al*. have also reported that ensuring compliance to school food policy in Samoa is challenging^(37)^. This presents an opportunity for sharing and reflecting on practice within the Pacific region and with the wider global school food community to identify strategies that may result in more manageable and sustainable monitoring and evaluation practices. This highlights the need to discuss how collaboration can be facilitated in this unique setting, with stakeholders from multiple sectors and diverse needs. However, this requires recognition of the significant geographic reach of the Pacific Islands and consideration of how collaboration can be supported. Stakeholders saw potential for more strategic collaborations; however, further work is needed to understand how to best support this in the region.

While we recognise that we have only captured the perspectives of the workshop participants, who may not have been able to answer all the questions posed and may have reflected personal views in the answers provided, this study includes informed stakeholders from eleven PICT, education and health sectors and a regional organisation providing a broad reach of perspectives. We believe the information captured through this study has not been published and this work provides an important contribution to advancing the dialogue of how to create healthy school food environments in this region. The unique setting of the Pacific Islands region gives rise to significant challenges, for example, geographical dispersion and lack of communication channels, that may not be seen elsewhere, highlighting the novelty of this information. The information provided by stakeholders only represents views at the time of the workshop, and these will likely change based on other internal/external events, that is, changes in government, climate events, etc., but this work provides an important foundation for discussion of the future of school food environments in the Pacific Islands region. We recognise the overlap between school food and nutrition education, school feeding programmes and school food activities and hence have used a definition of SFP that captures a range of activities, including school feeding programmes. This was presented to workshop attendees to ensure they understood this context. While recently developed, the FAO School Food and Nutrition Education framework^(22)^ provided a structure in which to frame discussions about SFP in this region.

## Conclusion

SFP should be considered as an opportunity to promote sustainable, healthy eating behaviours and improve health outcomes in the Pacific Islands region. Food provision and associated nutrition education in schools provides an opportunity to target students and the wider school community. There is a high level of motivation for SFP activities in the region; however, local food environments and a lack of strategic alignment of SFP to organisational priorities and policy, advocacy and organisational leadership, and opportunities for collaboration may be constraining the use of these programmes. Addressing these constraints and considering opportunities to integrate SFP into existing frameworks, a whole of community approach, mechanisms for increased collaboration, greater professional development and awareness activities (particularly for teachers), and improved monitoring and evaluation could improve awareness of SFP and enhance the use of SFP in the region.
